# Investigation of the Relationship between the Disease Severity and Quality of Life of Psoriasis Patients and Their Anthropometric Measurements and Diets

**DOI:** 10.3390/healthcare10112323

**Published:** 2022-11-20

**Authors:** Ali Timucin Atayoglu, Aslı Gizem Çapar, Eda Basmisirlioglu, Yagmur Yasar, Yusuf Aykemat, Ayten Guner Atayoglu, Neriman Inanc

**Affiliations:** 1 Department of Family Medicine, International School of Medicine, Istanbul Medipol University, Istanbul 34815, Turkey; 2 Department of Nutrition and Dietetic, Faculty of Health Science, Nuh Naci Yazgan University, Kayseri 38170, Turkey; 3 Department of Nutrition and Dietetic, Faculty of Health Science, Erciyes University, Kayseri 38280, Turkey; 4 Ataturk Family Medicine Center, Istanbul 34307, Turkey

**Keywords:** nutrition, quality of life, obesity, psoriasis, integrated healthcare, family physicians

## Abstract

Psoriasis is a multifaceted, chronic, inflammatory skin disease that impacts patients’ quality of life. The aim of this study was to investigate the relationship between the disease severity and quality of life of psoriasis patients and their nutritional status. The study included 40 patients in the psoriasis group, compared with 40 healthy individuals in the control group. A questionnaire for determination of socio-demographic characteristics and nutritional intake, anthropometric measurements, psoriasis area and severity index (PASI), and dermatology life quality index (DLQI) were used for the assessments. Daily food consumption details were recorded for three consecutive days to determine daily energy and nutrient intakes. Compared to the control group, the frequencies of single participants, primary school graduates, and housewives were higher in the psoriasis group (*p* < 0.05). The psoriasis patients weighed more and had an increased waist/hip ratio in comparison with the healthy individuals. The energy intake was lower in the psoriasis group (*p* < 0.01). There was not a significant statistical difference in the intake of proteins, fiber, vitamin A, vitamin E, vitamin C, Zn, Fe, and Mg supplements between the groups. However, there was an inverse correlation between the daily vitamin E intake and PASI scores (*p* < 0.05). There was a positive moderate correlation between the DLQI and PASI scores (*p* < 0.01). Our study indicated that lower daily vitamin E intake levels were associated with the severity of psoriasis. In addition to this, abdominal obesity seems to be another risk factor in psoriasis patients, even if they have a normal body mass index (BMI). An integrated healthcare approach with dermatologists, family physicians, and dietitians is essential to the management of psoriasis.

## 1. Introduction

Psoriasis is a complex, immune-mediated, chronic inflammatory skin disease, typically characterized by erythematous plaques with a silver scale [1]. The estimated prevalence of psoriasis ranges from 2% to 4% worldwide, and it affects both sexes equally [2,3].

Psoriasis is a chronic disease with a significant impact on quality of life [4]. Most psoriasis cases can be managed in the primary healthcare setting [2]; however, the management of severe cases is a challenge even for experienced dermatologists [3,4,5]. For treatment, patients’ conditions can be grouped into mild, moderate, and severe forms. Mild cases can often be treated with topical corticosteroids and emollients that include vitamin D analogs, or retinoids, while cases with severe forms may need phototherapy or systemic therapy with retinoids, methotrexate, cyclosporine, apremilast, or biologic immune-modifying agents [2,3,4,5]. At this juncture, a holistic approach is valuable due to the biopsychosocial nature of the disease, and integrated healthcare could make a contribution to the treatment of psoriasis. In addition to a number of pharmacologic therapies, non-pharmacologic approaches such as stress management, smoking and alcohol cessation, and weight control are important [2].

Nutritional status is an essential factor in the management of certain skin diseases; therefore, there is a growing interest in managing psoriasis through diet [6]. Some diets might have a positive impact on psoriasis because they tend to prevent inflammatory conditions in general [7,8]. Additionally, a number of studies suggest including vitamin supplements during psoriasis treatments, with limited amount of evidence supporting direct benefits [9,10]. Studies have reported that diets enriched with antioxidants, beta-carotene, vitamin-C, phenolics and flavonoids, which can be found in natural products, help to improve dermatological lesions [7,8,9,10]. Certain vitamins exhibit hydrophilic antioxidant activity, whereas others hinder lipid peroxidation from spreading. Hence, their use may benefit the treatment of psoriasis resulting from the ingestion of antioxidant agents. No specific nutritional therapy for psoriasis has been proven yet; however, a low-energy diet (of 800–1000 kcal/day) is recommended for overweight or obese patients [7,11]. 

Anthropometry has long been utilized for the assessment of nutritional status [12]. One of the clinical utilities of anthropometry is in defining obesity. In general, obesity—defined as a BMI ≥ 30 kg/m^2^ [6]—is used as an indicator of the body fat distribution [13]. However, the best measurement to define obesity has not been uniformly agreed upon, as is demonstrated in one study which compared body mass index (BMI), waist circumference, waist-to-hip ratio, and waist-to-height ratio [14]. Moreover, BMI has some limitations since it cannot differentiate between body fat and lean body mass, and it does not reflect the degree of body fat. Recently, it has been increasingly evident that central obesity, which reflects visceral adiposity, can be used as a better indicator of metabolic abnormalities and that waist circumference, which represents abdominal obesity, might be a better predictor of the risk of incident psoriasis in comparison to BMI [15]. Increasing epidemiological evidence suggests that obesity is associated with an increased risk of psoriasis [13]; the risk of psoriasis in obese people might be twice of that in individuals of a normal weight [15]. High BMI values may negatively affect the clinical manifestation of the disease in psoriasis patients [15,16,17,18]. Obesity is a chronic disease with a systemic inflammatory effect [17]. Furthermore, the efficacy of the treatment of psoriasis can be influenced by obesity [4].

Therefore, the aim of this study was to determine the relationship between the disease severity and quality of life of psoriasis patients and their nutritional status.

## 2. Methods and Materials

### 2.1. Study Design

Adult subjects (aged > 18 years) of both genders were included. Psoriasis patients were recruited from the outpatient clinic in the Dermatology Department of the Erciyes University Hospital in Kayseri, Turkiye. Healthy individuals were recruited from the hospital staff. Patients who were diagnosed based on the clinical and histopathological examination findings at least ten years ago and rely on topical treatment only were included in the study. Patients who were on systemic therapies, patients who were pregnant or breastfeeding, or patients with infectious diseases or malignancy, patients with mental health issues, additional non-psoriasis skin conditions, or other comorbidities were excluded from the study. 

All participants agreed to participate in the study between February 2019 and April 2019, giving both written and verbal consent. The sample size was calculated with the Minitab 19.2.0 software. The study included 40 psoriasis patients (psoriasis group) along with 40 randomly selected healthy participants (control group). A questionnaire evaluating socio-demographic characteristics and nutritional intake, anthropometric measurements, psoriasis area and severity index (PASI), and dermatology life quality index (DLQI) were used for the assessment of the relationship between the disease severity and quality of life of psoriasis patients and their nutritional status.

### 2.2. Anthropometric Measurements

Body weight was measured with a tolerance of 100 g. Height measurements were done without shoes by using a wall-mounted stadiometer. BMI was calculated, based on weight (kg)/ height^2^ (m^2^). The waist and hip circumferences were measured with a tolerance of 0.1 cm using an inflexible tape at the smallest circumference below the rib cage and above the umbilicus, and at the largest circumference between the waist and knees [19].

### 2.3. Questionary (Socio-Demographic Characteristics and the Nutrient Intake)

Participants were interviewed for about 8 to 10 min. Before the interview, each patient was informed in compliance with the structure of the standard interview. A questionnaire was used for data collection and the assessment of study parameters. Dietary intake was assessed with three-day food diaries, from which daily energy level and nutrient intakes were determined by using the Nutrition Information System (BeBiS) 7.0 computer package program developed at Hohenheim University, Stuttgart, Germany. The patients were informed of the food portions by using the Catalogue of Food and Nutrition [18]. 

### 2.4. Dermatology Life Quality Index (DLQI)

Dermatology life quality index (DLQI) is the most widely used quality of life scale specific to dermatological diseases. DLQI comprises a total of 10 questions about the patient’s feelings, daily activities, leisure time, school/work life, personal relationships, and treatment [20]. All of the questions have a one-week recall period. Each of the ten items of the questionnaire are rated on a 4-point scale (“not at all” or “not relevant” = 0, “a little” = 1, “a lot” = 2 and “very much” = 3), yielding a total score of 0 to 30. A higher total score represents a greater impairment of HRQoL [20].

### 2.5. Psoriasis Area Severity Index (PASI)

Psoriasis area severity index (PASI) is the most commonly used clinical scoring system to determine the severity of psoriasis [20]. In the current study, a PASI score of less than 7 was considered to indicate mild disease, a score between 7 and 12 was considered moderate, and a score over 12 was considered severe [21,22].

### 2.6. Statistical Analysis

The data were analyzed using SPSS Statistics V22.0 (IBM Corp., Chicago, IL, USA). The data was expressed as mean, numbers, and percentage. The Chi-square test was performed to determine the difference between categorical variables. The Mann–Whitney U and Spearman correlation tests were used for the evaluation of numerical data. A *p* value of <0.05 was considered statistically significant for all analyses.

## 3. Results

Among the participants, 23 (57.5%) of the patients were males and 17 (42.5%) were females (Table 1). The mean age at the onset of the disease was 18.61 ± 13.96 years, and patients and controls were matched for age in Table 2.

Both the weight and the waist/hip ratio levels were higher in the psoriasis group compared with the control group (*p* < 0.05 for both). Although the study did not include any patients with a BMI value of >30 kg/m^2^, values for both the BMI and the circumferences of the waist and hip were higher in the psoriasis group when compared to the control group (*p* < 0.001). While the energy intake was lower in the psoriasis group compared to the control group (*p* < 0.01), no statistically significant differences were found between the groups regarding the intakes of proteins, fiber, vitamin A, vitamin E, vitamin C, Zn, Fe, and Mg supplements. In the psoriasis group, the frequency of alcohol consumption was 5%; in both groups smoking had a frequency of 42.5% (*p* > 0.05) (Table 2).

In the psoriasis group, 28 (70%) of the patients had PASI scores less than 7, and 12 (30%) of the patients had PASI scores of 7 to 14, as determined by the PASI scores of 3.28 ± 2.75 (mean) and 2.4 (median). The body weights of the patients had a weak negative correlation with the DLQI and PASI scores (*p* < 0.05). There was a positive moderate correlation between the DLQI and PASI scores (*p* < 0.01). Moreover, there was an inverse relationship between the daily vitamin E intake and PASI scores (*p* < 0.05) (Table 3). 

## 4. Discussion

In the current study, 70% of the psoriasis patients had a mild, and 30% of the patients had a moderate, form of psoriasis. We found a higher frequency of psoriasis in male patients. However, similar to previous studies, our collected PASI scores demonstrated no relationship between gender and disease activity [23]. In comparison with healthy individuals, alcohol consumption rate was not higher among the psoriasis patients, and no statistically significant differences were found between the groups regarding smoking. However, it should be noted that previous studies have shown that alcohol consumption may facilitate the development of psoriasis, especially in men with a family history of psoriasis [24]. Significant improvements in the PASI scores have been reported in the literature for patients who adopt a low-energy diet for 4 to 8 weeks compared to those eating a normal diet [7]. However, a recent review reported that dietary manipulation alone may not cause a large effect on psoriasis patients but may be a potential adjunct to the treatment [25].

Previously, obesity in psoriasis patients was linked to social isolation, poor eating habits, depression, low levels of physical activity, and high alcohol consumption [26]. It was established that obesity is an inflammatory condition, and that fat tissue can act like either an immune or endocrine organ [27]. It has been suggested that adipokines secreted by the adipose tissue are involved in the pathogenesis of comorbidities associated with metabolic syndrome and psoriasis [28]. Although the mechanism has not fully been explained yet, it has been suggested that pro-inflammatory mediators released from adipocytes in obese patients aggravate psoriasis [15]. Obese patients have elevated inflammatory cytokines, such as IL17 and IL23, which are involved also in the pathogenesis of psoriasis [6,25], and it is hypothesized that the added inflammatory burden of obesity may increase the severity of psoriasis in adult patients [17]. Recent studies have reported a bidirectional association between psoriasis and obesity [29].

Abdominal obesity seems to be another factor in increasing the risk of psoriasis. Several studies have indicated that “abdominal obesity” may be a more reliable measure than BMI in predicting health risks [30,31]. The risk of psoriasis was shown to be higher in men with abdominal obesity, even if they had normal BMI levels [15]. Consistent with the previous studies, values of weight, waist/hip ratio, BMI, waist circumference, and hip circumference were higher in the psoriasis group compared to the control group [32].

Jensen et al. demonstrated that a low-energy diet contributed both to reducing lesions and even improving the DLQI [33]. Gisondi et al. reported that in obese patients, a 5–10% body mass reduction improved the response to treatment [34]. Therefore, patients that follow dietary recommendations may decrease the dosage of medications. Furthermore, a low-energy diet may contribute to prolonged remission of symptoms [35]. In patients with a low-energy diet, greater relief of symptoms was observed in comparison with the group that only underwent medical treatment [33]. In our study, we used the DLQI to measure the health-related quality of life of patients, and the relationship of the DLQI scores to PASI scores, anthropometric measurements, and food consumption were evaluated. The DLQI and PASI scores yielded parallel results in the assessment of psoriasis severity and quality of life. However, contrary to expectations, quality of life improved with high body weights. We observed that quality of life deteriorated as the severity of the disease increased. Although discussable, that is consistent with the opinion that extra weight may have positive effects on quality of life in people with some diseases [36]. Zawisza et al. demonstrated that weight loss was associated with better quality of life in healthy individuals, while the reverse was observed in individuals with a chronic disease, whose weight loss might be an indicator of poorer quality of life. Therefore, they suggested an inverse U-shaped association between BMI and quality of life [37]. 

In the current study, there was a negative correlation between the daily intake of vitamin E and the PASI scores. It has been suggested that high levels of vitamin E consumption may affect the disease severity positively [9]. Previously, a meta-analysis linked severity of psoriasis to low serum levels of vitamin E [38]. Oxidative stress, and reactive oxygen species can lead to damage to the vascular endothelial cells; this increases the permeability of small vessels and the transmission of inflammatory cells, which may aggravate the inflammation in psoriasis. Therefore, psoriasis patients are recommended to consume food with large amounts of antioxidants, particularly vegetables, for protection against the harmful effects of free radicals. Antioxidants may suppress secretion of TNF-α, while their deficiency may be a potential risk factor for the development of psoriasis [7,39]. Studies provide sound evidence for the immunoregulatory role of vitamin E [40]. The possible mechanisms may be performed both directly through alterations of cell membrane function and cell signaling pathways, or indirectly via modulation of inflammatory mediators, including the production of PGE2 and cytokines [40,41]. In addition to the T-cell function and modulation of Th1 response, it is possible that vitamin E can regulate other types of immune cells, causing improved immune response and decreased risk of immune-mediated diseases [40]. Comprehension of the impact and the mechanisms of vitamin E is crucial to determining ideal vitamin E intake in psoriasis patients.

This study had some limitations. First, the sample size could be regarded as a relatively small one. Second, it is a possibility that, during documentation of the nutrient intake, the patients might have tendency to be more careful than usual to receive a better-quality diet; thus, the information about their nutrient intake may not reflect their dietary routine. Third, our study did not include any patients with a BMI value of >30 kg/m^2^. While BMI > 30 was not our target population in this study, it would be interesting to check the impact of vitamin E-rich diet on this group of patients who have BMI >30. Another limitation is the fact that the patient group included no one with a severe form of the disease that may need to receive systemic therapies. By the same token, no patient who was being treated with systemic treatments was included in the study, which limits our conclusions only to the mild to moderate forms of psoriasis. Therefore, a long-term and more comprehensive future study with a larger sample size, full range of BMI values and PASI scores seems quite an acceptable proposition to expand and verify our conclusions. 

## 5. Conclusions

Anthropometric measurements in the psoriasis patients manifest higher values when compared to the control group. Meanwhile, abdominal obesity seems to be another risk factor in psoriasis patients, even if the patients have normal BMI levels. The current study also indicated that lower daily vitamin E intake levels were associated with the severity of psoriasis, in accordance with earlier studies that showed negative correlation between serum vitamin E levels and disease progression. Vitamin E is known to modulate immunity because of its protective effect against oxidation of poly-unsaturated fatty acids in the membranes of immune cells, opening them up to oxidative damage. While future studies may reveal the potential mechanism associated with the protective effects of vitamin E, relationship between the prognosis of psoriasis and antioxidants needs to be clarified as well. A holistic and proactive approach, including a healthy diet, is essential in the management of psoriasis. Therefore, an integrated healthcare approach characterized by a high degree of collaboration among health professionals, dermatologists, family physicians, and dietitians is essential in the management of psoriasis. 

## Figures and Tables

**Table 1:** Comparison of socio-demographic characteristics of the groups.

Characteristics	Psoriasis Group n (%)	Control Group n (%)	Total	*c* ^2^	*p*
**Gender**					
**Male**	23 (57.5)	23 (57.5)	46 (57.5)	<0.0001	1.000
**Female**	17 (42.5)	17 (42.5)	34 (42.5)		
**Marital Status**					
**Married**	7 (17.5)	19 (47.5)	26 (32.5)	8.205	0.004
**Unmarried**	33 (82.5)	21 (52.5)	54 (67.5)		
**Educational Status**					
**Primary School**	15 (37.5)	8 (20.0)	23 (28.8)	10.136	0.038
**High School**	8 (20.0)	13 (32.5)	21 (26.2)		
**Graduate**	10 (25.0)	17 (42.5)	27 (33.8)		
**Postgraduate**	2 (5.0)	2 (5.0)	4 (5.0)		
**Others**	5 (12.5)	0 (0.0)	5 (6.2)		
**Occupation**					
**State officer**	3 (7.5)	6 (15.0)	9 (11.2)	12.551	0.028
**Worker**	5 (12.5)	6 (15.0)	11 (13.8)		
**Self-employment**	10 (25.0)	2 (5.0)	12 (15.0)		
**Retired**	5 (12.5)	4 (10.0)	9 (11.3)		
**Housewife**	11 (27.5)	6 (15.0)	17 (21.2)		
**Others**	6 (15.0)	16 (40.0)	22 (27.5)		
**Smoking**					
**Yes**	17 (42.5)	17 (42.5)	34 (43.5)	<0.0001	1.000
**No**	23 (57.5)	23 (57.5)	46 (57.5)		
**Alcohol Consumption**					
**Yes**	2 (5.0)	3 (7.5)	5 (6.2)	0.213	0.644
**No**	38 (95.0)	37 (92.5)	75 (93.8)		

**Table 2:** Comparison of groups’ age, anthropometric measurements, daily energy intake, some macro and micronutrients.

	Psoriasis Group	Control Group	*Z*	*p*
	Mean ± Sd	Mean ± Sd		
**Age (year)**	40.20 ± 14.02	39.60 ± 15.57	0.327	0.743
**Weight (kg)**	79.49 ± 16.69	70.20 ± 13.70	−2.181	0.029
**BMI (kg/m^2^)**	28.10 ± 04.83	24.12 ± 3.86	−3.700	<0.0001
**Waist Circumflex (cm)**	98.10 ± 14.39	85.30 ± 12.82	−3.794	<0.0001
**Hip Circumflex (cm)**	106.75 ± 12.60	98.50 ± 8.56	−3.401	<0.0001
**Waist/Hip ratio**	0.90 ± 0.12	0.86 ± 0.01	−2.184	0.029
**Energy (kcal)**	1239.33 ± 616.53	1605.50 ± 486.44	−2.829	0.005
**Protein (g)**	74.92 ± 38.09	62.27 ± 16.61	−1.891	0.059
**Fiber (g)**	23.34 ± 8.42	17.69 ± 7.75	−1.838	0.066
**Vitamin A (µg)**	1072.31 ± 847.64	813.70 ± 516.46	−1.482	0.138
**Vitamin E (mg)**	17.61 ± 10.70	15.21 ± 8.19	−0.813	0.416
**Vitamin C (mg)**	75.81 ± 68.54	71.50 ± 60.75	−0.082	0.935
**Zinc (mg)**	11.90 ± 5.44	08.96 ± 03.42	−1.564	0.118
**Iron (mg)**	10.22 ± 4.12	09.86 ± 03.10	−1.660	0.097
**Magnesium (mg)**	244.57 ± 95.71	215.85 ± 67.45	−1.188	0.235

**Table 3:** The relationship between DLQI, PASI scores and anthropometric measurements, energy and nutrient intake of patients with psoriasis.

	DLQI Score		PASI Score		Weight		BMI		Energy	
	r	*p*	r	*p*	r	*p*	r	*p*	r	*p*
**DLQI score**	-	-	0.468	0.002	−0.264	0.049	−0.141	0.387	0.220	0.895
**PASI score**	0.468	0.002	-	-	−0.316	0.047	0.168	0.299	−0.076	0.641
**Weight**	−0.264	0.049	−0.316	0.047	-	-	0.781	<0.0001	0.314	0.049
**BMI**	−0.141	0.387	0.168	0.299	0.781	<0.0001	-	-	0.122	0.453
**Waist circumference**	−0.1933	0.232	−0.1753	0.279	0.8184	0.001	0.818	0.001	0.2983	0.007
**Waist/Hip**	0.0876	0.591	−0.03	0.854	0.3278	0.003	0.3056	0.006	0763	0.501
**Energy (kcal)**	0.220	0.895	−0.076	0.641	0.314	0.049	0.122	0.453	-	-
**Protein (g)**	−0.71	0.665	0.087	0.595	0.395	0.012	0.160	0.324	0.760	<0.0001
**Fiber (g)**	−0.028	0.863	−0.055	0.735	0.303	0.057	0.061	0.709	0.566	<0.0001
**Vitamin A (µg)**	0.128	0.430	0.102	0.532	0.116	0.487	0.243	0.131	0.288	0.072
**Vitamin E (mg)**	−0.093	0.593	−0.346	0.029	0.117	0.473	0.188	0.247	0.335	0.034
**Vitamin C (mg)**	−0.176	0.277	−0.047	0.772	0.132	0.418	0.141	0.285	0.287	0.073
**Iron (mg)**	−0.079	0.762	−0.098	0.548	0.328	0.039	0.117	0.473	0.671	<0.0001
**Zinc (mg)**	−0.097	0.552	−0.152	0.349	0.511	0.001	0.308	0.053	0.694	<0.0001
**Magnesium (mg)**	−0.022	0.891	0.021	0.898	0.245	0.128	0.136	0.402	0.504	0.001

## Data Availability

Data will be available from the corresponding author upon reasonable request.

## References

[1] Papp K.A., Gniadecki R., Beecker J., Dutz J., Gooderham M.J., Hong C.-H., Kirchhof M.G., Lynde C.W., Maari C., Poulin Y. (2021). Psoriasis Prevalence and Severity by Expert Elicitation. Dermatol. Ther..

[2] Lim W., How C., Tan K. (2021). Management of psoriasis in primary care. Singap. Med. J..

[3] Damiani G., Bragazzi N.L., Aksut C.K., Wu D., Alicandro G., McGonagle D., Guo C., Dellavalle R., Grada A., Wong P. (2021). The Global, Regional, and National Burden of Psoriasis: Results and Insights From the Global Burden of Disease 2019 Study. Front. Med..

[4] Colombo D., Perego R. (2013). Quality of Life in Psoriasis. Expert Review of Pharmacoeconomics & Outcomes Research.

[5] Lebwohl M., Thaçi D., Warren R. (2021). Addressing challenges associated with long-term topical treatment and benefits of proactive management in patients with psoriasis. J. Eur. Acad. Dermatol. Venereol..

[6] Kanda N., Hoashi T., Saeki H. (2020). Nutrition and Psoriasis. Int. J. Mol. Sci..

[7] Garbicz J., Całyniuk B., Górski M., Buczkowska M., Piecuch M., Kulik A., Rozentryt P. (2021). Nutritional Therapy in Persons Suffering from Psoriasis. Nutrients.

[8] Rocha A.C.L., Bortoletto M.C., da Costa A.C., Oyafuso L.K.M., Sanudo A., Tomita L.Y. (2021). Low serum lycopene, and adequate α-tocopherol levels in patients with psoriasis: A cross-sectional study. Nutr. Health.

[9] Yazdanpanah M.J., Vahabi-Amlashi S., Nematy M., Shaelaei N., Mohajeri S.A.R., Tafazzoli Z. (2021). Association of Serum Lipid Profiles and Dietary Intakes of Vitamin E and Fiber with Psoriasis Severity. Caspian J. Int. Med..

[10] Stanescu A.M.A., Simionescu A.A., Diaconu C.C. (2021). Oral Vitamin D Therapy in Patients with Psoriasis. Nutrients.

[11] Barrea L., Megna M., Cacciapuoti S., Frias-Toral E., Fabbrocini G., Savastano S., Colao A., Muscogiuri G. (2020). Very low-calorie ketogenic diet (VLCKD) in patients with psoriasis and obesity: An update for dermatologists and nutritionists. Crit. Rev. Food Sci. Nutr..

[12] Bhattacharya A., Pal B., Mukherjee S., Roy S.K. (2019). Assessment of nutritional status using anthropometric variables by multivariate analysis. BMC Public Health.

[13] Snekvik I., Smith C.H., Nilsen T.I., Langan S.M., Modalsli E.H., Romundstad P.R., Saunes M. (2017). Obesity, Waist Circumference, Weight Change, and Risk of Incident Psoriasis: Prospective Data from the HUNT Study. J. Investig. Dermatol..

[14] Kidy F., Dhalwani N., Harrington D.M., Gray L.J., Bodicoat D.H., Webb D., Davies M., Khunti K. (2017). Associations Between Anthropometric Measurements and Cardiometabolic Risk Factors in White European and South Asian Adults in the United Kingdom. Mayo Clin. Proc..

[15] Han J.H., Lee J.H., Han K.D., Kim H., Bang C.H., Park Y.M., Lee J.Y., Kim T.-Y. (2019). Increased risk of psoriasis in subjects with abdominal obesity: A nationwide population-based study. J. Dermatol..

[16] Pirro F., Caldarola G., Chiricozzi A., Burlando M., Mariani M., Parodi A., Peris K., de Simone C. (2021). Impact of Body Mass Index on the Efficacy of Biological Therapies in Patients with Psoriasis: A Real-World Study. Clin. Drug Investig..

[17] Fleming P., Kraft J., Gulliver W.P., Lynde C. (2015). The Relationship of Obesity With the Severity of Psoriasis. J. Cutan. Med. Surg..

[18] Rakicioglu N., Tek N., Ayaz A., Pekcan G. (2017). Yemek ve Besin Fotoğraf Kataloğu. Ölçü ve Miktarlar.

[19] Hammond K.A., Mahan L.K., Raymond J.L. (2012). Dietary and Clinical. Krause’ s Food & the Nutrition Care Process.

[20] Rencz F., Szabó Á., Brodszky V. (2021). Questionnaire Modifications and Alternative Scoring Methods of the Dermatology Life Quality Index: A Systematic Review. Value Health.

[21] Warren R., Hansen J., Reich K., Paul C., Puig L. (2020). Complete clearance and psoriasis area and severity index response for brodalumab and ustekinumab in AMAGINE-2 and -3. J. Eur. Acad. Dermatol. Venereol..

[22] Goel S., Bansal S., Chopra D., Batra J. (2021). Metabolic Derangements in Patients of Psoriasis and Their Association with Psoriasis Area Severity Index Score: A Cross-Sectional Study. Natl. J. Lab. Med..

[23] Hägg D., Sundström A., Eriksson M., Schmitt-Egenolf M. (2017). Severity of Psoriasis Differs Between Men and Women: A Study of the Clinical Outcome Measure Psoriasis Area and Severity Index (PASI) in 5438 Swedish Register Patients. Am. J. Clin. Dermatol..

[24] Svanström C., Lonne-Rahm S.-B., Nordlind K. (2019). Psoriasis and alcohol. Psoriasis Targets Ther..

[25] Pona A., Haidari W., Kolli S.S., Feldman S.R. (2019). Diet and psoriasis. Dermatol. Online J..

[26] Nowowiejska J., Baran A., Grabowska P., Lewoc M., Kaminski T.W., Flisiak I. (2021). Assessment of Life Quality, Stress and Physical Activity Among Patients with Psoriasis. Dermatol. Ther..

[27] Alexaki V. (2021). The Impact of Obesity on Microglial Function: Immune, Metabolic and Endocrine Perspectives. Cells.

[28] Hao Y., Zhu Y.-J., Zou S., Zhou P., Hu Y.-W., Zhao Q.-X., Gu L.-N., Zhang H.-Z., Wang Z., Li J. (2021). Metabolic Syndrome and Psoriasis: Mechanisms and Future Directions. Front. Immunol..

[29] El-Boghdady N.A., Ismail M.F., Abd-Alhameed M.F., Ahmed A.S., Ahmed H.H. (2018). Bidirectional Association Between Psoriasis and Obesity: Benefits and Risks. J. Interf. Cytokine Res..

[30] Powell-Wiley T.M., Poirier P., Burke L.E., Després J.-P., Gordon-Larsen P., Lavie C.J., Lear S.A., Ndumele C.E., Neeland I.J., Sanders P. (2021). Obesity and Cardiovascular Disease: A Scientific Statement From the American Heart Association. Circulation.

[31] Di Jiang D., Wang L., Bai C., Chen O. (2019). Association between abdominal obesity and asthma: A meta-analysis. Allergy Asthma Clin. Immunol..

[32] Acar E.M., Ilter N., Elbeg Ş. (2019). Association of leptin, resistin, and high-molecular-weight adiponectin levels with psoriasis area and severity index scores, obesity, and insulin resistance in psoriasis patients. Dermatol. Sin..

[33] Jensen P., Zachariae C., Christensen R., Geiker N., Schaadt B.K., Stender S., Hansen P.R., Astrup A., Skov L. (2013). Effect of Weight Loss on the Severity of Psoriasis: A Randomized Clinical Study. JAMA Dermatol..

[34] Gisondi P., Del Giglio M., Di Francesco V., Zamboni M., Girolomoni G. (2008). Weight loss improves the response of obese patients with moderate-to-severe chronic plaque psoriasis to low-dose cyclosporine therapy: A randomized, controlled, investigator-blinded clinical trial. Am. J. Clin. Nutr..

[35] HYGEIA Public Health—Article. http://www.h-ph.pl/hyg.php?opc=AR&lng=en&art=634.

[36] Napoli N., Shah K., Waters D.L., Sinacore D.R., Qualls C., Villareal D.T. (2014). Effect of weight loss, exercise, or both on cognition and quality of life in obese older adults. Am. J. Clin. Nutr..

[37] Zawisza K., Tobiasz-Adamczyk B., Galas A., Jabłońska K., Grodzicki T. (2019). Changes in Body Mass Index and Quality of Life—Population-Based Follow-up Study COURAGE and COURAGE-POLFUS, Poland. Appl. Res. Qual. Life.

[38] Liu X., Yang G., Luo M., Lan Q., Shi X., Deng H., Wang N., Xu X., Zhang C. (2021). Serum vitamin E levels and chronic inflammatory skin diseases: A systematic review and meta-analysis. PLoS ONE.

[39] Sumathi S., Babu S.V., Karthikeyan K. (2019). Evaluation of vitamin D status, selenium and C-reactive protein level in psoriasis. Int. J. Res. Dermatol..

[40] Lewis E.D., Meydani S.N., Wu D. (2018). Regulatory role of vitamin E in the immune system and inflammation. IUBMB Life.

[41] Wu D., Meydani S.N. (2008). Age-associated changes in immune and inflammatory responses: Impact of vitamin E intervention. J. Leukoc. Biol..

